# What Do We Know About the Drivers of Health and Equity? A Narrative Review of Graphic Representations

**DOI:** 10.1089/heq.2020.0013

**Published:** 2020-10-19

**Authors:** Marjory L. Givens, Bridget B. Catlin, Sheri P. Johnson, Elizabeth A. Pollock, Victoria N. Faust, Paula Tran Inzeo, David A. Kindig

**Affiliations:** Population Health Institute, School of Medicine and Public Health, University of Wisconsin-Madison, Madison, Wisconsin, USA.

**Keywords:** social determinants of health, determinants of equity, graphic representations

## Abstract

**Purpose:** Frameworks can be influential tools for advancing health and equity, guiding population health researchers and practitioners. We reviewed frameworks with graphic representations that address the drivers of both health and equity. Our purpose was to summarize and discuss graphic representations of population health and equity and their implications for research and practice.

**Methods:** We identified publicly available frameworks that were scholarly or practice oriented and met defined inclusion and exclusion criteria. The identified frameworks were then described and coded based on their primary area of focus, key elements included, and drivers of health and equity specified.

**Results:** The variation in purpose, concepts, drivers, underlying theory or scholarly evidence, and accompanying measures was highlighted. Graphic representations developed over the last 20 years exhibited some consistency in the drivers of health; however, there has been little uniformity in depicting the drivers of equity, disparities or interplay among the determinants of health, or transparency in underlying theories of change.

**Conclusion:** We found that current tools do not offer consistency or conceptual clarity on what shapes health and equity. Some variation is expected as it is difficult for any framework to be all things to all people. However, keeping in mind the importance of audience and purpose, the field of population health research and practice should work toward greater clarity on the drivers of health and equity to better guide critical analysis, narrative development, and strategic actions needed to address structural and systemic issues perpetuating health inequities.

## Introduction

In the early 1990s, two broad conceptual frameworks for considering the factors that influence health were published.^1,2^ These two examples of frameworks continue to guide the work of population health researchers and practitioners. Graphic representations, such as conceptual frameworks, are often used to help clarify and distill a set of concepts that are fundamental and their relationship to a phenomenon. Conceptual frameworks are particularly important when a single theory, theoretical framework, or perspective is deemed insufficient to capture a complex phenomenon. In research, conceptual frameworks shape the epistemological paradigm—or belief systems for how and why we know the world—brought to the phenomena of interest. Conceptual frameworks can help guide the framing of research projects, how relevant questions are determined, how meaning is attached to data, and the resulting development of research findings. Similarly, in practice, such frameworks can help shape how people consider and approach the identification and prioritization of problems to be addressed, identify and interpret relevant data and research findings, and implement and evaluate solutions. Such frameworks, therefore, have the potential to affect how we collectively understand, investigate, and address societal and community issues.

Population health is one such complex phenomenon for which both researchers and practitioners have developed and graphically represented frameworks. The graphic representations of these frameworks show a broad picture of health and its drivers that go beyond simply defining health as the absence of disease and health care as the primary driver of health. A collection of these frameworks, representing a continuously expanding set of determinants that influence health, was reviewed in 2015 by the Canadian Council on Social Determinants of Health (CCSDH).^3^

Many of the frameworks in the CCSDH review do not, however, specifically address the factors that influence how determinants of health and their related health outcomes are distributed within and across communities (or populations). We define such factors that shape the distribution of determinants and, subsequently, health outcomes as drivers of equity. As McCartney et al.^4^ noted, scholars have offered multiple definitions of health equity over the years. For the purposes of this review, we follow Whitehead's 1992 definition: “Equity in health implies that ideally everyone should have a fair opportunity to attain their full health potential and, more pragmatically, that none should be disadvantaged from achieving this potential.”^5^ More recently, new frameworks and associated graphic representations of health have emerged that do attempt to incorporate drivers of equity in the context of health, such as the World Health Organization's (WHO's) Commission on the Social Determinants of Health (CSDH) conceptual framework^6^ and the Bay Area Regional Health Inequity Initiative (BARHII).^7^ We find such frameworks to be important to a field that increasingly prioritizes examination of the distribution of determinants and, subsequently, health outcomes across communities. We therefore set out to review such frameworks.

Similar in approach to the CCSDH review, this essay discusses graphic representations of frameworks of drivers of health and equity. It differs from the CCSDH review in that it centers inclusion criteria on the drivers of equity (or inequity or inequalities). Given the focus on graphic representations, it does not review an exhaustive catalog of frameworks used to explain drivers of health, nor does it include frameworks that represent drivers of equity outside a health context. Our emphasis on the drivers of health and equity is also different from another Canadian review by the National Coordinating Center for Determinants of Health, which focused on knowledge to action models.^8^ In addition, it is not designed as a formal evaluation of the frameworks reviewed. Rather, the purpose of this review is to stimulate discussion on how frameworks (describing relevant concepts and their relationships for study of a topic^9^) or models (descriptions or analogies used to help visualize something that cannot be directly observed^10^) shape understanding of knowledge and principles used in population health research and practice and how we collectively understand the drivers of health and equity in our work.

## Methods

Our approach for this work went beyond what is normally applied for a traditional narrative review (see, e.g., the University of Alabama-Birmingham's comparison of systematic and narrative reviews), particularly around inclusion/exclusion criteria, which are seldom reported for narrative reviews. Our approach also differed from other systematic and narrative reviews in that we were searching for graphical depictions rather than original research studies. So, to identify existing frameworks that address the drivers of health and equity, our initial search strategy involved using Google, PubMed, and Google Scholar from March 2019 to January 2020 for English-language documents and images based on the following keywords: (determinants of health OR wellbeing) AND (health equity OR health inequalities) AND (frameworks OR models OR theories). We followed up with snowball sampling from citations of documents found and searches of relevant organizational websites, reports, and gray literature were also reviewed. We conducted a broad, but not exhaustive, search for frameworks that were published in the 21st century when materials became more widely available on the internet. We did not focus our search on formal research databases because we were particularly interested in finding images of frameworks that have been used in practice.

From these materials, we selected frameworks for review that were scholarly or practice oriented and met the following inclusion criteria:

- Must mention equity or like terms (inequity, inequality, disparities, and differences across groups): for example, several well-known or emerging frameworks do not mention equity or like terms.^1,2,11–14^
- Must include a graphical depiction of the drivers of health equity: for example, Braveman has written extensively about health equity, but we were unable to find a conceptual framework in her work. Similarly, the new Well-being in the Nation Measures^15^ did not include a graphic at the time of retrieval.
- Must include specific drivers of health equity (e.g., education and employment): for example, Chang^16^ only lists general drivers of equity (e.g., opportunities and barriers).
- Must include arrows, nesting, sequencing, or other graphical approaches to show drivers' relationships to equity: for example, King County's 14 Determinants of Health Equity^17^ provides a comprehensive listing of determinants (or drivers) of health equity, but does not show how they relate to equity, health, or each other.
- Must address general health/equity, not specific topics such as drivers of asthma, behavioral medicine, and gender equity: for example, Gee and Payne-Sturges's framework^18^ is specific to environmental health disparities.
- Must focus on frameworks addressing drivers of health/equity rather than frameworks focused on actions to improve health/equity: for example, the frameworks reviewed by Davison et al.^8^

Frameworks were excluded from the sample if they were not posted on the internet or if they represented derivatives of included frameworks. For example, several organizations have published modified versions of the WHO CSDH^4^ and BARHII^7^ frameworks. Inclusion criteria were developed collaboratively among the authors, based on disciplinary and practical knowledge. Results are expected to inform discussion in the field on what shapes health and equity, how it is graphically portrayed, and the implications for our research and practice.

Once we had selected frameworks to be included in our review, one co-author extracted information directly from applicable articles or websites and another co-author checked the extraction to be sure we accurately recorded information about the frameworks. Following discussions with all the authors that led to the development of a list of key background information and elements, we then conducted a qualitative review of the frameworks with two co-authors coding information independently and then reconciling differences in interpretation of frameworks and background information. The lead author served as the final arbiter for any difference not reconciled. Our intent was to be sure that we reported shared, rather than individual, interpretations of information reflected in the frameworks. We coded the following information:

- Primary area of focus:
- ○ Community practice (local health assessment and improvement)
- ○ Policy development (supporting national or state decision making), and/or
- ○ Research.
- Key elements:

What is in the framework:

1. Role of individuals and communities in driving health and equity (CCSDH also captured this element in their review^3^)
2. Identifies fundamental causes of inequity
3. Mentions multiple disparity domains (e.g., race and SES).

How do things relate:

4. Shows interactions between determinants (CCSDH also captured this element in their review^3^)
5. Recognizes inequities in determinants and/or policies (as well as outcomes)

Salience, credibility, and legitimacy:

6. Based on peer-reviewed literature (e.g., material supporting the framework includes citations)
7. Recognition of importance of upstream action (CCSDH also captured this element in their review^3^)
8. Includes some metrics.

Given that graphical depictions of frameworks are frequently designed to be used outside the accompanying text and so often stand alone, or in isolation of context, our qualitative review involved coding frameworks without consulting accompanying text. The only exceptions made were for elements 6 and 8. As noted above, three of the key elements were drawn from the CCSDH review. The remaining key elements were identified based on discussion among the co-authors about potentially desirable criteria for a framework depicting the drivers of health and equity.

After this qualitative review, we captured and coded the drivers of health and equity listed in each framework. Two co-authors performed the coding and then discussed and reconciled the initial results together. All the co-authors met to review and finalize the coding schema. Drivers were coded into major categories and subcategories to tally counts within each grouping.

## Results

We found 27 health and equity frameworks that met our inclusion and exclusion criteria, described in Table 1. These frameworks are listed in chronological order with the year of initial publication, names of the authoring individuals or organizations, framework or article title, country of origin, stated purpose and target audience, stated context and origins (with underlying explicit or implicit theory if clearly articulated), and source.

**Table 1.** Overview of Selected Frameworks

Years	Organization or author(s)	Framework or article title	Country of origin	Stated purpose and target audience	Context and origins (theory if clearly articulated)
2001	Diderichsen et al.^32^	The social basis of disparities in health	Multiple	Written as a chapter in a book “designed to bring the research and policy analysis of the Global Health Equity Initiative to a wider nonspecialist readership of students, professionals, and policymakers”	Epidemiology-based framework for understanding social origins of health inequities with four main mechanisms (social stratification, differential exposure, differential susceptibility, and differential consequences) that play a role in generating health inequities
2001	Starfield^33^	Improving equity in health: a research agenda	United States	Published in an academic journal with a stated purpose to “define equity in a way that makes it easier to recognize, consider its origin and causes, and discuss the imperative for research to provide some alternatives for reducing and eliminating it”	Justice. Defines “equity in *health* is the absence of systematic differences in one or more aspects of health status across socially, demographically, or geographically defined populations or population subgroups” and “Equity in *health services* implies that there are no differences in health services where health needs are equal (horizontal equity) or that enhanced health services are provided where greater health needs are present (vertical equity)”
2003	Community Guide (Anderson et al.^24^)	Social environment and health model	United States	As part of the systematic review process for addressing the topic “the social environment,” the Task Force on Community Preventive Services developed a conceptual framework “to illustrate the entire public health context in which interventions might take place”	The Task Force used the framework to generate a list of candidate interventions to promote healthy social environments
2004	Schulz and Northridge^19^	Social determinants of health and environmental health promotion	United States	The stated goal of the academic article containing this framework is to “contribute to the conceptualization of social determinants of health” and in “understanding how social and environmental inequalities contribute to health disparities”	Draws on disciplines of sociology and environmental and social epidemiology. Derived from a conceptual model developed by Schulz et al. (2002)^35^ on racial and spatial relationships as determinants of health, modified based on Northridge and Sclar^36^ work linking urban planning and public health
2008	CDC Promoting Health Equity (Brennan Ramirez et al.^21^)	Pathways from Social Determinants to Health (adapted from BCBS Minnesota and Anderson et al.^21^)	United States	Published in a workbook “for public health practitioners and partners interested in addressing social determinants of health to promote health and achieve health equity.” “Appreciation of how societal conditions, health behaviors, and access to health care affect health outcomes can increase understanding about what is needed to move toward health equity”	Published as part of “Promoting Health Equity-A Resource to Help Communities Address Social Determinants of Health” with content drawn from a forum sponsored by CDC in 2003. The framework was adapted from those prepared by Blue Cross and Blue Shield of Minnesota Foundation and Anderson et al.^21^ Principles of social justice influence “multiple interactions and the resulting health outcomes: inequitable distribution of social determinants contributes to health disparities and health inequity, whereas equitable distribution of social determinants contributes to health equity”
2010	BARHII^7^	A public health framework for reducing health inequities	United States	This conceptual framework “illustrates the connection between social inequalities and health, and focuses attention on measures that have not characteristically been within the scope of public health department epidemiology. This framework has been used widely as a guide to health departments undertaking work to address health inequities. It has been formally adopted by the California Department of Public Health as part of their decision-making framework”	The BARHII is a coalition of the San Francisco Bay Area's 11 public health departments committed to advancing health equity with its origins going back to the mid-1990s. BARHII became an organization in 2002, initially focusing on nutrition and physical activity, but then decided picking a single focus violates one of the first premises of working with communities that require collaborative decision making on priorities. BARHII conducts activities to increase understanding of the link between public health and social justice
2010	CSDH, WHO (Solar and Irwin^6^)	Multiple frameworks: Figure 2: Structural determinants: the social determinants of health inequities Figure 3: Intermediary determinants of health Figure 4: Summary of the mechanisms and pathways represented in the framework Figure 5: Final form of the CSDH conceptual framework	Multiple^a^	The Commission's purpose was to revive the understanding of health as a social phenomenon and address WHO's constitutional commitments to health equity and social justice. The Commission sought to find “answers to three fundamental problems: 1) where do health differences among social groups originate, if we trace them back to their deepest roots; 2) what pathways lead from root causes to the stark differences in health status observed at the population level; and 3) in light of the answers to the first two questions, where and how should we intervene to reduce health inequities?” Their purpose was to define an action-oriented framework to identify policies to tackle SDH	Drawing on the principles of human rights, the CSDH reviewed three types of theories: (1) psychosocial approaches, (2) social production of disease/political economy of health, and (3) ecosocial frameworks and the main pathways to explain causation: (1) social selection/mobility, (2) social causation, and (3) life course perspectives. They defined core values of health equity, human rights, and distribution of power
2010	CRSIHS (Borrell et al.^37^)	Conceptual framework of the determinants of health inequalities	Spain	Published in an academic journal, this framework was developed because “policy interventions to reduce health inequalities need to be planned with clarity based on comprehensive macro-to-micro theoretical models that provide a positive heuristic to address why, how, and where particular interventions would make a difference in health inequalities, as well as who should be targeted to receive interventions”	Adopted by the SOPHIE Project, aimed “to generate new evidence on the impact and effectiveness of policies in tackling health inequalities and to develop innovative methodologies for the evaluation of these policies in Europe.” The framework was adapted from the CSDH's framework to fit the European context
2010	WHO (Blas and Kurup^38^)	Conceptual framework for understanding health inequities, pathways, and entry points	Multiple^a^	Developed as a way of taking the work of the WHO CSDH and translating it into concrete, workable actions. Intended audience is policymakers and program managers	Developed as part of the work of the WHO Priority Public Health Conditions Knowledge Network. The framework is included in the Cardiovascular Diseases chapter, but has applicability to many other conditions
2011	HI DOH Chronic Disease Management and Control Branch^39^	Root causes of health disparities in chronic disease in Hawaii	United States	Part of the Chronic Disease Disparities Report 2011: Social Determinants, which “compiles surveillance information and other data sources on chronic disease disparity issues” specific to Hawaii's population. Stated purpose of the report: “(1) provide a broad picture of some of the health disparities and social determinants of health that are apparent across all chronic diseases, risk factors, and risk markers in Hawaii and (2) illustrate that these differences follow a social gradient, not just ‘high’ or ‘low’ differences in population groups.” Intended audience: decision makers, community-based organizations, and the public	The framework “depicts the traditional Hawaii Ahupua‘a concept wherein resources and capacity flow from Mauka (the mountain) to Makai (the sea), from ‘upstream’ to ‘downstream.’” “The depiction of Ahupua‘a, resources and capacity to sustain the community flow from the mountain to the sea where health and well-being are sustained in a healthy, holistic cycle”
2011	Prevention Institute^40^	THRIVE clusters and factors	United States	THRIVE provides “a framework for understanding how structural drivers play out at the community level to impact community determinants, and consequently, health and safety outcomes, and inequities in outcomes.” Intended audience: community members and practitioners	“THRIVE was created to answer the question: what can be done in communities to improve health and safety and reduce inequities?” THRIVE includes a framework and a tool for “engaging community members and practitioners in assessing the status of community determinants, prioritizing them, and taking action to change them to improve health, safety, and health equity.” It was originally developed in 2002–2003 and then updated in 2010–2011
2012	VA DOH^41^	Health equity and social determinants of health framework	United States	The framework is part of the 2012 Virginia Health Equity Report, intended for two broad target audiences: “1) public health and medical professionals interested in data to guide their grant programs and interventions and 2) policy makers, advocates, and communities interested in … solutions to creating communities that provide equitable opportunities to be healthy”	Part of the 2012 Virginia Health Equity Report as a “resource of relevant and useful information regarding the health status of disadvantaged populations across the state. This information serves as a baseline from to develop new plans and strategies with our Commonwealth partners.” Based on an ecosocial approach and “identifies specific areas of focus that are necessary for success—individual behavior change, enhancing health promoting SDOH, and assuring that all Virginians have equitable access to these SDOH regardless of socioeconomic status, race/ethnicity, gender, place of residence, etc.”
2013	NHS Scotland (Beeston et al.^42^)	Health inequalities: theory of causation	Scotland	The framework is part of a review conducted to inform the 2013 Scottish Ministerial Task Force on Health Inequalities. The framework “provides a visual representation of the current understanding on the causes of health inequalities and how these are manifested as differences in mortality, morbidity, well-being, and healthy life expectancy”	The review was commissioned by the Scottish Parliament Health and Sport Committee following the release of a report on health inequalities (by Audit Scotland), which found that national policies and strategies had shown limited evidence of impact. The aim of the report was to assess whether the then current strategy (*Equally Well)* was “effective and what else might be needed” and complemented a stakeholder engagement process. The framework is based on multiple theories of causation: fundamental causes; social, economic, and physical environment; and individual experiences. There are also two summary versions of the framework.
2013	WRHA^23^	Framework for understanding and addressing health equity	Canada	Intent of report was to “lay a foundation upon which we can collectively build Winnipeg's health equity action plan” and “to facilitate collaborative conversations … toward achieving greater health equity in Winnipeg.” It recognizes that “actions needed to achieve health for all do not lie solely, or even primarily, within the health care sector, we are all in this together”	The framework is part of a report that “represents the work and consensus of working groups under the direction of the Winnipeg Health Region Promoting Health Equity Oversight Committee.” The framework was based on scan of published health equity literature (academic and gray) to find reports with relevance to Winnipeg. The framework was developed based on over 1000 recommendations from these reports that were then coded and pooled
2015	Colorado Department of Public Health and Environment^25^	Health equity: an explanatory model for conceptualizing the social determinants of health	United States	This framework is part of the 2015–2019 Colorado State Health Improvement Plan. Intended audience is public health system partners and the public. The plan is “intended to guide health improvement work throughout (the) state.” The framework is “a visual model for conceptualizing the broad, complex, and interrelated determinants of health”	Developed by the Colorado Department of Public Health and Environment (and promoted nationally by ASTHO). Includes recognition of life stages
2015	Harris County Public Health, TX^43^	Health equity framework	United States	The framework is part of Harris County's Health Equity Policy that is intended “to serve as guidance for the procedural guidelines designed to help institutionalize health equity goals into Harris County Public Health & Environmental Services programs, policies, services, and interventions.” Intended audience is state government public health staff	No specific theory mentioned, but acknowledges the County Health Rankings percentage of health outcomes attributable to social determinants of health (social and economic opportunities and physical conditions). Note: This spherical framework includes “actions to break the cycle” of inequity as wells as drivers of health and health inequity
2015	NIA (Hill et al.^44^)	NIA health disparities research framework	United States	The framework was developed “to assess progress and opportunities toward stimulating and supporting rigorous research to address health disparities.” Its development “was considered important for identifying gaps, opportunities, and progress in health disparities research at the NIA”	A task force from the NACA launched “a review of NIA's health disparities research portfolio and researcher training activities for diverse investigators.” The results of this review prompted a recommendation that the NIA develop an integrative, conceptual framework to guide health disparities research
2015	NIMHD (Alvidrez et al.^24^)	The NIMHD research framework	United States	This article presents the NIMHD research framework as “a tool for conceptualizing and depicting the wide array of determinants that promote or worsen minority health or cause, sustain, or reduce health disparities.” The “framework can be used to assess minority health and health disparities research, as well as priorities for the future”	According to the authors, the NIMHD “framework reflects a hybrid of two existing models: the NIA health disparities research framework and the socioecological model”
2015	PHAC^20^	Toward health equity: a tool for developing equity-sensitive public health interventions	Canada	PHAC “developed this practice tool for public health professionals to support them in developing equity-sensitive public health interventions.” Note: Although it is focused on interventions, it also contains information on drivers of inequities	Draws on the work of Whitehead, Braveman, and Solar and Irwin. Focuses on conditions that contribute to health inequity, identifying entry points to address health equity, mediating factors, engagement strategies, and equity targeting
2015	RWJF (Plough et al.^45^)	Culture of health action framework	United States	The framework “serves two purposes: to catalyze a broad social movement by offering entry to diverse individuals, organizations, and sectors; and to guide RWJF's own role and place among many others in this movement.” The Framework was “developed in collaboration with the RAND Corporation to chart and measure the nation's progress in achieving improved population health, well-being, and equity”	“The Framework condenses extensive research and practical community experience into four interconnected Action Areas. Each represents significant strategic opportunities to realize the vision of a Culture of Health at the national and community level. The Outcome Areas represent the improvements we expect to see as the nation makes progress across the four action areas. Each action area has three Drivers, which are priority areas for attention and innovation needed to make the ongoing systemic, cultural, and social changes required”
2015	Victorian Health Promotion Foundation (VicHealth^22^)	Fair foundations: the VicHealth framework for health equity	Australia	VicHealth developed this framework as a conceptual and planning tool to guide action on the social determinants of health inequities. Intended audience appears to be public health professionals	The VicHealth framework for health equity draws on the conceptual framework developed by WHO drawing on the following approaches: psychosocial; social production of disease/political economy of health; and ecosocial frameworks
2017	NASEM: Communities in Action: Pathways to Health Equity^46^	A conceptual model for community-based solutions to promote health equity	United States	This framework comes from the report of the NASEM Committee on Community-Based Solutions to Promote Health Equity in the United States. This conceptual model grounded the work of the Committee	The charge of the committee was “to review the state of health disparities and explore the underlying conditions and root causes that contribute to health inequity to inform much-needed efforts to reverse such inequities.” The conceptual model was informed by the RWJF's Culture of Health Framework and the Prevention Institute's *Countering the Production of Health Inequities: A Framework of Emerging Systems to Achieve an Equitable Culture of Health* (2016)
2017	PHAC (*Pan-Canadian Health Inequities Data Tool*^48^)	Pan-Canadian Health Inequalities Data Tool	Canada	This “framework” is a hierarchical map of available indicators in the Pan-Canadian Health Inequalities Data Tool addressing inequalities in health status and health determinants	In 2012, Canada, along with other WHO Member States, endorsed the Rio Political Declaration on SDH, pledging to take action to promote health equity (defined by the WHO as “the absence of avoidable or remediable differences among groups of people”). Strengthening the capacity to monitor and report on health inequalities was recognized as a critical foundation for achieving meaningful progress toward this goal
2018	PAHO^42^	The PAHO equity commission's conceptual framework	Multiple^b^	The PAHO Equity Commission has identified substantial social and economic inequalities within and between countries in the Americas. The Commission also identified “climate change, environmental threats, relationship with land, and the continuing impact of colonialism, racism, and the history of slavery as critical factors.” Its aim is “to provide a better understanding of these challenges as well as make proposals for effective action to address them”	“The framework is based on the (WHO) CSDH conceptual framework, but goes beyond it in important ways: there is emphasis on structural racism, colonialism, and importance of relationships to land; … greater emphasis on the environment and climate change; there is a more explicit focus on human rights; and greater emphasis on inequities according to gender, ethnicity, sexual orientation, life stage, and disability (and their interrelations)”
2019	ChangeLab Solutions^49^	5 Fundamental Drivers of Health Inequity	United States	This framework is part of *A Blueprint for Changemaker: Achieving Health Equity Through Law and Policy*, which “presents legal strategies and best practices to help policymakers and communities improve health outcomes…for changemakers—people who have seen the effects of inequality and are ready for a new approach that will ensure that everyone has what they need to be as healthy as possible”	Referencing the work of the WHO CSDH, ChangeLab Solutions states that their experience and “portfolio of practice throughout the nation as well as research evidence point to five fundamental drivers of health inequity that are created by and therefore are amenable to legal and policy interventions”
2019	Dover and Belon^50^	The Health Equity Measurement Framework (HEMF)	Canada	Dover and Belon “propose an expanded, more descriptive framework to better capture the complexity of SDOH on the generation of health (in)equity.” The HEMF “is designed to describe the multitude of SDOH in a causal framework and guide the quantitative analysis of health equity for ongoing public health surveillance and policy development”	“The process of developing the HEMF was based on a framework synthesis, which involved integrating existing frameworks, narrative literature review, and consultation with potential knowledge users. The cornerstone of the HEMF is the well-known WHO's Commission on Social Determinants of Health conceptual framework”
2019	NIMHD (Duran and Perez-Stable^51^)	Relationship between health determinants and health disparity outcomes	United States	In an article titled “Novel Approaches to Advance Minority Health and Health Disparities Research,” the authors present a framework that “illustrates health determinants as contributing factors on the adverse health disparity outcomes”	The authors propose a new definition of health disparity: “a health difference that adversely affects defined disadvantaged populations, based on one or more health outcomes.” While they recognize the importance of social determinants, they suggest that additional health determinants need to be considered to understand the contributors of health disparities

^a^ The WHO CSDH includes commissioners from the following countries: United Kingdom (chair), Australia, Canada, Chile, China, Egypt, Iran, Italy, India, Japan, Kenya, Mozambique, Senegal, Sweden, Tanzania, and the United States.

^b^ The Commission of the PAHO on Equity and Health Inequalities includes members from the following countries: United Kingdom (chair), Argentina, Brazil, Bolivia, Canada, Ecuador, Jamaica, and the United States.

ASTHO, Association of State and Territorial Health Officials; BARHII, Bay Area Regional Health Inequities Initiative; BCBS, Blue Cross Blue Shield; CDC, Centers for Disease Control and Prevention; CRSIHS, Commission to Reduce Social Inequalities in Health in Spain; CSDH, Commission on the Social Determinants of Health; HEMF, Health Equity Measurement Framework; HI DOH, Hawai'i State Department of Health; NACA, National Advisory Council on Aging; NASEM, National Academies of Sciences, Engineering, and Medicine; NHS, National Health Service; NIA, National Institute on Aging; NIMHD, National Institute of Minority Health and Health Disparities; PAHO, Pan-American Health Organization; PHAC, Public Health Agency of Canada; RWJF, Robert Wood Johnson Foundation; SDH, Social Determinants of Health; SDOH, Social Determinants of Health; SOPHIE, Structural Policies for Health Inequalities Evaluation; THRIVE, Tool for Health and Resilience in Vulnerable Environments; VA DOH, Virginia Department of Health; WHO, World Health Organization; WRHA, Winnipeg Regional Health Authority.

The year of initial publication of the frameworks reviewed ranged from 2001 to 2019. Of those identified, more frameworks (seven) were published in 2015 than in any other year. Sixteen of the frameworks (59%) were developed in the United States, with the remaining frameworks coming from Australia, Canada, and Europe. Eight of the frameworks were published in peer-reviewed literature with the remaining 19 (70%) coming from gray literature. More than half of the frameworks were published by government entities (national, regional, or local). Eight of the frameworks were either developed by the WHO's Commission on Social Determinants or were influenced by their work; and of these, all but one was developed outside the United States. While few of the framework descriptions clearly articulated their underlying theories, the theories mentioned in the WHO work included ecosocial, psychosocial, and social production of disease/political economy of health. Other theories mentioned included fundamental causation and life course. Principles of social justice and human rights were also mentioned in a minority of the framework descriptions.

Tables 2 and 3 provide the results of our qualitative review of the 27 frameworks. With their concentration in the scholarly and gray literature, we identified frameworks with a variety of primary areas of emphasis, the most common of which was policy development. The early frameworks (from 2001 to 2004) that we found were intended to guide policy development or research. The first frameworks for community practice did not appear until 2008. Many of the frameworks intended to guide research did not appear until 2015.

**Table 2.** Primary Area of Focus of Selected Frameworks

Years	Organization or author(s)	Framework	Community practice	Policy development	Research
2001	Diderichsen et al.^32^	The social basis of disparities in health		×	
2001	Starfield^33^	Improving equity in health: a research agenda			×
2003	Community Guide^34^	Social environment and health model		×	
2004	Schulz and Northridge^19^	Social determinants of health implications for environmental health promotion			×
2008	CDC Promoting Health Equity^21^	Pathways from social determinants to health	×		
2010	BARHII^7^	A public health framework for reducing health inequities	×		
2010	WHO CSDH^6^	Multiple frameworks		×	
2010	CRSIHS^37^	Conceptual framework of the determinants of health inequalities		×	
2010	WHO^38^	Conceptual framework for understanding health inequities, pathways, and entry points		×	
2011	HI DOH^39^	Root causes of health disparities in chronic disease in Hawaii	×		
2011	Prevention Institute^40^	THRIVE factors	×		
2012	VA DOH^41^	Health equity and social determinants of health framework	×	×	
2013	NHS Scotland^42^	Health inequalities: theory of causation	×	×	
2013	WRHA^23^	Framework for understanding and addressing health equity	×	×	
2015	CO DPH^25^	Health equity: an explanatory model for conceptualizing the social determinants of health	×	×	
2015	Harris County Public Health, TX^43^	Health equity framework		×	
2015	NIA^44^	NIA health disparities research framework			×
2015	NIMHHD^24^	The NIMHHD research framework			×
2015	PHAC^20^	Toward health equity: a tool for developing equity-sensitive public health interventions	×	×	
2015	RWJF^45^	Culture of health action framework		×	×
2015	Victorian Health Promotion Foundation (VicHealth)^22^	Fair foundations: the VicHealth framework for health equity	×	×	
2017	NASEM: Communities in Action: Pathways to Health Equity^46^	A conceptual model for community-based solutions to promote health equity	×	×	
2017	PHAC^47^	Pan-Canadian Health Inequalities Data Tool	×		×
2019	PAHO^48^	The PAHO Equity Commission's Conceptual Framework		×	×
2019	ChangeLab Solutions^49^	5 Fundamental drivers of health inequity	×	×	
2019	Dover and Belon^50^	The Health Equity Measurement Framework (HEMF)		×	×
2019	NIMHHD^51^	Relationship between health determinants and health disparity outcomes			×
		Totals	13	17	9

CO DPH, Colorado Department of Public Health.

**Table 3.** Key Elements of Selected Frameworks

Years	Organization or author(s)	Role of individuals and communities	Identifies fundamental causes of inequity	Mentions multiple disparity domains	Shows interactions between determinants	Recognizes inequities in determinants and/or policies	Based on peer-reviewed literature	Importance of upstream action	Includes some metrics	Total
2001	Diderichsen et al.^32^					×	×			2
2001	Starfield^33^				×		×	×	×	4
2003	Community Guide^34^				×		×	×		3
2004	Schulz and Northridge^19^	×	×		×	×	×	×	×	7
2008	CDC^21^	×		×	×	×	×		×	6
2010	BARHII^7^	×		×	×	×		×		5
2010	CSDH^6^			×	×	×	×	×	×	6
2010	CRSIHS^37^		×	×	×		×	×		5
2010	WHO^38^				×	×	×	×	×	5
2011	HI DOH^39^		×	×			×	×	×	5
2011	Prevention Institute^40^	×		×		×	×		×	5
2012	VA DOH^41^	×		×		×			×	4
2013	NHS Scotland^42^	×	×			×	×		×	5
2013	WRHA^23^						×			1
2015	CO DPH^25^								×	1
2015	Harris County, TX^43^		×	×		×		×	×	5
2015	NIA^44^		×	×			×			3
2015	NIMHHD^24^	×		×			×		×	4
2015	PHAC^20^	×	×	×		×	×	×	×	7
2015	RWJF^45^						×	×	×	3
2015	VicHealth^22^	×		×		×	×	×	×	6
2017	NASEM^46^	×	×			×	×	×		5
2017	PHAC^47^			×		×			×	2
2019	PAHO^48^			×	×	×	×	×		5
2019	ChangeLab^48^		×			×	×	×		4
2019	Dover and Belon^50^				×		×		×	3
2019	NIMHHD^51^						×			1
	Total	10	9	14	10	16	22	15	17	
	Percent	37%	33%	52%	37%	59%	81%	56%	63%	

Two-thirds of the frameworks were framed positively, that is, their outcomes were stated in terms of well-being or equity. Outcomes in the remaining one-third of the frameworks were framed negatively, comprising terms such as inequity or disparity.

We found that at least 22 (or 81%) frameworks drew from peer-reviewed literature—this was the most common key element identified among the frameworks. The next most frequent key element was the inclusion of some metrics, which we found for 17 (63%) of the frameworks. Examples of metrics include measures such as age-adjusted mortality rates, percent of population under the federal poverty level, and the Gini coefficient as an income inequality metric. Beyond acknowledging that there are inequities in health outcomes, over half of the frameworks also recognized the existence of inequities in determinants or policies (16 total or 59%) or highlighted the importance of upstream action (15 total or 56%). Just over half of frameworks reviewed acknowledged multiple disparity domains (14 total or 52%), such as among racial/ethnic groups, socioeconomic status, or by age groups.

The least frequently classified key elements were highlighting the roles of individuals and communities (found in only 37% of frameworks), interaction between determinants (37%), and identifying fundamental causes of inequity (33%).

Frameworks developed to serve as a guide to future research are likely to be subject to different criteria than frameworks designed to guide community practice. Thus, given the different purposes of the frameworks, it is not surprising that no single framework included all the key elements. The frameworks with the most key elements was Schulz and Northridge's,^19^ which includes all the key elements, except for mentioning multiple disparity domains, and the Public Health Agency of Canada's,^20^ which includes all elements, except for showing interactions between the determinants of health and equity. Three frameworks include all but two key elements: Centers for Disease Control and Prevention's Promoting Health Equity^21^ (missing were identifying fundamental causes of inequity and the importance of upstream actions), the WHO CSDH frameworks^6^ (missing were role of individuals and communities and identifying fundamental causes), and Victorian Health Promotion Foundation's framework^22^ (missing identifying fundamental causes of inequity and showing interactions between determinants).

Three frameworks included only one of the key elements we identified: the Winnipeg Regional Health Authority^23^ and the National Institute of Minority Health and Health Disparities^24^ frameworks, which were both based on peer-reviewed literature, and the Colorado Department of Public Health framework,^25^ which included some metrics.

In Table 4, we display a summary of the various drivers, sometimes termed determinants, of health and equity found in the frameworks reviewed. We listed drivers that were found in at least three of the frameworks in bold text. At the highest level, we coded drivers that addressed (1) individual characteristics, (2) community conditions and community context, (3) societal context, and (4) overarching drivers, that is, which can occur at the individual, community, or societal level. Within each of these five categories, we identified up to six subcategories of drivers of health and equity. The subcategories and drivers are listed in alphabetical order.

**Table 4.** Drivers of Health and Equity in Selected Frameworks

Category	Subcategory	Drivers of health and equity
Individual characteristics	Awareness, beliefs, and worldview	Attitudes; Bias; Knowledge; Mindset and expectations; **Religion and religious beliefs**
	Biology and genetics	**Age;** Cholesterol and blood pressure
	Health behaviors	**Alcohol and drugs (substance abuse); Diet, dietary practice, overeating, and poor nutrition; Low physical activity and physical inactivity**; Personal health practices; **Sexual behavior; Smoking and tobacco use**
	Individual experience, identity, and social position	Burden of housework and caring; Caregiver-child interaction; **Disability**; **Discrimination—interpersonal**; Discrimination response; Experience of class, racism, gender, and immigration; Exposure to community illness; Exposure to health-promoting/health-harming factors; **Exposure to toxins and pathogens (pollutants, noise, damp, mold, and lead);** Family functioning and microbiome; **Gender**; Health care—receipt of needed care; Health care—consumer experience and treatment preferences; Health care—patient-clinician relationship; Housing and material assets; **Illiteracy/literacy**; **Immigration status;** Individual health education; **Limited English**; Need, **Numeracy**; **Race and ethnicity;** School readiness; **Sexual orientation; Social and socioeconomic class, position, and location**; Specific exposure
	Individual experience and life course	Childhood; Cognitive development (early); Early child development; Intrauterine life (adverse); Working life
	Psychosocial, risk and resilience, and sense of belonging	Control; **Coping factors (active coping, problem solving, stress management, cognitive reframing, and emotional regulation);** Health beliefs; Health screening and seeking care when needed; Identity-cultural; Loneliness; Optimism and pessimism; Potential to fully participate in society; Resilience and coping mechanisms; Self-concepts; Sense of community; Sense of personal security and belonging; **Stress—family, financial, and occupational**
	Socioeconomic	Education level; **Employment status; Income and financial situation (individual);** Insecurity—financial; Job security and control; **Living and working conditions; Material circumstances; Occupation (individual);** Opportunity—Individual, family, and disparities
Community conditions and context	Community assets	Corporations and businesses and corporate practices**; Education and learning opportunities—distribution and quality**; **Food, healthy food availability, and food security**; Government agencies; Not-for-profit organizations; Opportunities for learning and developing capacity; Protection, police services, police response; Public health services**;** Resources-financial**;** Resources-human**;** Resources-material**;** Resources-natural; Resources-social**; Retail businesses; Safety**; Safety net services; Sanitation; **Schools—availability, quality, and environment**; Social and cultural institutions; **Social protection;** Social services
	Community assets—health care	**Health care—access; Health care-access, utilization and quality;** Health care-barriers to care; Health care—provider availability; **Health insurance affordability and quality;** Health promotion, disease and injury prevention; **Health system characteristics (integration);** Immunization
	Characteristics of assets/services	Acceptability; Accessibility**;** Accountability; Affordability**;** Appropriateness; **Availability;** Comprehensiveness; Continuity; Effectiveness; **Environment—service (accessibility, affordability, availability, navigability, and quality);** Sustainability; Universality
	Environment—built or natural	**Air, water, and soil; Environmental and physical characteristics and quality;** Gentrification and displacement; **Housing—quality and crowding**; **Land use**; **(Safe) Leisure and recreation opportunities;** Look, feel (esthetics)**; Neighborhood living conditions and environment;** Parks and open space; Residential environment; **Transportation and getting around**
	Environment—economic and employment	Affordability and standard of living in communities (rent, fuel, etc.); Community development, investment and employment opportunities; Economic development; Economic opportunity; Education—availability and affordability beyond K12; **Employment and working opportunity distribution and work environment;** Employment opportunities—distribution; Environmental—economic; Income inequality; **Income and wealth (living wages and local wealth)**; **Occupational hazards; Poverty;** Standard of living; Wealth (material)—distribution; Work—financial and other benefits; Work/job availability
	Environment—social, political, cultural, and history	Community capacity; Community reentry; Crime/Criminalization; **Environment—social and cultural; Geographical, place, territory and political factors; Governance—governance that limits meaningful participation;** Government and political tradition; Marginalization; Partnerships—number and quality; Political structures**; Residential segregation**; Social mobility; **Social stratification, structure, and hierarchy; Socioeconomic and political context**; Strategic partnerships; **Stressors;** Unfair treatment; Urban, urbanization and rural; **Violence and anger (neighborhood)**
Societal context	Civic muscle	Advocacy; Civic engagement and participation; Community capacity building; Community organizing; Community and civic engagement and involvement; Democratic engagement and representation; Democratization; Governance; Knowledge; Participation; People; Policy that support collaboration; **Social capital, cohesion, connectedness, civic engagement, collective efficacy, inclusion; Social integration, networks, participation, support, and trust**
	Crisis and chronic conditions	Climate change; Deprivation; Discrimination—structural; Herd immunity; Migration; **Racism-institutional, structural, racial barriers, and enablers;** Reconciliation
	Environment—economic and employment	**Economy, economic conditions, global economic forces and economic systems and order; Labor market**
	Environment—social, political, and cultural	**Culture and history and values; Culture—ads/media;** Macrosocial factors; **Policy and political context;** Collective responses; Globalization; **Historical conditions, health disadvantage, and legacy (trauma)**
	Policies and law	**Laws and regulations zoning and enforcement; Macroeconomic policies;** Policy—economic; Policy—environmental; **Policy—health; Policy—public; Policy—social;** Policy—workplace; Public practices and spending; **Policy and policy entry points**
	Societal values and norms	Equity and equity objectives; Health as shared value; Health equity as shared vision and value; Ideologies; **Norms (social, economic, and legal), attitudes, culture, customs, principles, processes, traditions;** Social justice; Stereotypes; Structural bias; **Values (societal);** Willingness to act for common good
Overarching	Fundamental drivers of inequities	**Differential consequences; Differential exposure; Differential vulnerability;** Equitable access to SDOH by race, class, gender, residence, etc.; Intersectionality; Prejudice; Prestige; Stigma; Unequal distribution of factors
	Resources and allocation	Distribution of protections, rights and benefits; Equitable access to SDOH by race, class, gender, residence, etc.; Political influence and its distribution; Political priorities and decisions; **Power and disparities in political power;** Power—institutional; Technology
	Rights	Human rights; Indigenous rights; Treaty rights

*Note:* Drivers in bold were found in three or more frameworks.

In Table 5, we display the assigned categories and subcategories for the drivers included in each of the frameworks reviewed. Eleven of the frameworks listed drivers of health and equity that covered all four categories (subcategories): individual characteristics (awareness, beliefs, and worldview; biology and genetics; health behaviors; individual experience, identity, and social position; individual experience and life course; psychological, risk and resilience, and sense of belonging; and socioeconomics), community conditions and context (community assets; community assets-health care; characteristics of assets/services; environment built/natural; environment-economic, employment; environment-social, political, and cultural, history), societal context (civic muscle; crisis and chronic conditions; environment-economic, employment; environment-social, political, and cultural; policies and law; and societal values and norms), and overarching drivers (fundamental drivers of inequities; resources and allocation; and rights). Community conditions and context was the only category where all frameworks included at least one driver. The overarching category was the least common category, with only 15 frameworks including at least one driver.

**Table 5.** Assigned Subcategories of Drivers of Health and Equity in Selected Frameworks

		Individual characteristics							Community conditions and context			Community conditions and context			Societal context						Overarching		
Years	Organization or author(s)	Awareness/beliefs/worldview	Biological and genetics	Health behaviors	Individual experience/identity/social position	Individual experience—life course	Psychosocial/risk, resilience/sense of belonging	Socioeconomic	Community assets	Community assets— health care	Characteristics of assets/services	Environment—built or natural	Environment—economic and employment	Environment—social, political, cultural, and history	Civic muscle	Crisis and chronic conditions	Environment—economic/employment	Environment—social, political, and cultural	Policies and law	Societal values/norms	Drivers of inequities—fundamental	Resources and allocation	Rights
2001	Diderichsen et al.^32^	0	0	0	1	0	0	0	0	0	0	0	0	1	0	0	0	1	1	0	1	0	0
2001	Starfield^33^	0	0	0	0	0	0	0	1	1	0	1	0	0	0	0	0	1	1	0	0	0	0
2003	Community Guide^34^	0	0	0	0	0	0	0	1	1	0	1	1	1	1	0	1	1	0	1	0	1	0
2004	Schulz and Northridge^19^	0	0	1	1	0	1	1	1	1	1	1	1	1	1	1	1	1	1	1	1	1	1
2008	CDC^21^	0	0	0	0	0	0	0	0	0	0	1	0	0	0	0	1	0	0	0	0	0	0
2010	BARHII^7^	0	0	1	1	0	0	1	1	1	1	1	1	1	1	0	0	1	1	0	0	0	0
2010	CSDH^6^	0	0	0	1	0	0	1	1	1	0	1	0	1	1	0	1	1	1	0	1	1	0
2010	CRSIHS^37^	0	1	0	1	0	0	1	1	0	0	1	1	1	0	0	1	1	1	0	0	1	0
2010	WHO^38^	0	0	0	1	1	0	0	0	0	0	1	1	1	0	0	0	1	0	0	1	0	0
2011	HI DOH^39^	0	1	1	1	0	0	1	1	1	0	1	1	1	0	1	0	1	0	0	0	0	0
2011	Prevention Institute^40^	0	0	0	1	0	0	0	1	0	0	1	1	1	1	0	0	1	0	1	0	0	0
2012	VA DOH^41^	0	1	1	1	0	0	0	1	0	0	1	1	0	1	0	0	0	0	0	0	1	0
2013	NHS Scotland^42^	0	0	1	1	1	1	1	1	0	1	1	1	1	1	0	0	0	0	1	1	1	0
2013	WRHA^23^	0	0	0	0	1	0	1	1	1	1	0	1	1	1	1	1	0	0	1	0	0	1
2015	CO DPH^25^	0	1	1	1	0	0	1	1	1	0	1	1	1	1	1	0	0	0	0	0	1	0
2015	Harris County, TX^43^	1	0	1	1	0	1	1	1	1	1	1	1	1	0	0	0	1	1	0	1	1	0
2015	NIA^44^	1	0	1	1	0	1	1	0	1	0	0	1	1	1	1	0	1	0	1	1	0	0
2015	NIMHHD^24^	0	0	0	1	0	1	0	1	1	1	1	1	1	1	1	0	0	1	1	0	0	0
2015	PHAC^20^	0	0	1	1	0	1	1	1	0	0	0	0	1	1	1	0	1	1	1	0	1	0
2015	RWJF^45^	1	0	0	1	0	1	0	0	1	0	1	1	1	1	0	0	0	1	1	0	0	0
2015	VicHealth^22^	1	0	0	1	1	0	1	1	1	0	1	0	1	1	0	0	1	0	1	0	0	0
2017	NASEM^46^	0	0	0	0	0	0	1	1	1	0	1	1	1	1	0	0	0	0	1	0	0	0
2017	PHAC^47^	0	0	0	0	1	0	0	1	1	0	1	1	1	0	0	0	0	0	0	0	0	0
2019	PAHO^48^	0	0	0	1	1	0	1	1	0	0	1	0	1	0	1	1	1	0	0	1	0	0
2019	ChangeLab^49^	0	0	0	0	0	0	0	0	0	0	0	1	1	0	1	0	0	0	0	0	1	0
2019	Dover and Belon^50^	0	0	0	1	0	1	1	1	0	1	0	0	1	0	0	0	1	1	0	0	0	0
2019	NIMHHD^51^	1	0	1	1	0	1	1	1	1	1	1	1	1	0	1	0	1	0	0	0	0	0
	Total	5	4	10	20	6	9	16	21	16	8	21	19	24	15	10	7	17	11	11	8	10	2

*Note:* 1=Subcategory included in framework.

0=Subcategory not included in framework.

The most frequent subcategories identified were environment-social, political, and cultural (under community context, included in 24 out of 27% or 89% of frameworks), community assets and environment—built or natural (under community context, each included in 78% of frameworks), and individual experience, identity, and social position (under individual characteristics, included in 74% of frameworks).

## Discussion

We believe that frameworks can serve many purposes: they can inform research agendas, planning and decision making, or assessment. Frameworks can be considered boundary-spanning tools to engage new audiences among disciplines, sectors, or community groups. For example, the County Health Rankings measurement framework has helped advance conversation about the social determinants of health.^12^ Frameworks are also intended to organize thinking and shape narratives, which in turn shape the boundaries of what is possible for action. And frameworks can also be used to help raise awareness and make sense of the issues that shape health and equity.

We found many more frameworks than the 27 frameworks that met our criteria. The low number was both a reflection of our search approach and the stringency of our selection criteria. Several published frameworks that we found, but did not include, are derivatives of other frameworks and, as such, did not meet our inclusion criteria for review.

In terms of purpose, the focus of frameworks that we found from outside the United States was more likely to be on policy development. And, of the key elements, the non-United States frameworks were more likely to mention multiple disparity domains and recognize inequities in determinants and policies. However, the real benefit of including frameworks from outside the United States was the additional richness provided by the types of drivers of health and equity that they included.

Only eight of the frameworks were published in peer-reviewed publications and research was the stated purpose of most of these frameworks, that is, none of the eight were intended to guide community practice. However, we were heartened to see that many of the frameworks intended to guide community practice found in the gray literature were based on peer-reviewed literature. The linkage between scholarly work and practice continues to be important.

Recent dialogue about equity in the field of population health has focused on the importance of an asset-oriented approach, or positive framing. Asset-oriented approaches serve to counter the negative default assumptions about communities that bear disproportionate burden of inequitable conditions. However, it is not clear whether this approach is more likely to change the way equity is studied or practiced. Most of the frameworks reviewed were positively framed, none provided comprehensive metrics that quantify assets that influence health or measurement of equitable outcomes—a recognized current limitation in the field.^26^ For those positively framed, it is also unclear how to deal with historical policy and practice that underlie inequity, such as assimilation laws aimed at Native Americans and Jim Crow laws. Furthermore, some (positively or negatively framed) frameworks illustrate what should be measured, while others only illustrate what can currently be measured. For example, several frameworks include constructs that should be measured, such as civic engagement or political factors, whereas, in reality, what can be measured may be limited to metrics such as voter participation.

Given that equity is a comparative principle, or judgment about how a person or group of people is situated relative to others, it is intriguing that very few, if any, frameworks were explicit about underlying theories, values, and norms that provide context for sense making of what is avoidable, unfair, and unjust. The absence of clear guidance about whether and how conditions are unjustly produced, raises questions about utility for deriving innovative scholarship or practice.

As the field embraces intersectionality, frameworks will also need to evolve to acknowledge interactions among determinants and interconnections of multiple disparity domains. Few frameworks effectively did so among those we reviewed. While identifying many of the known relationships and interactions between components of a framework is important to guide future research, showing all such relationships in a framework intended to guide community practice can be confusing, sometimes described as arrow soup. Conversely, failing to show important interactions can lead to misunderstanding at the least, or far worse, could lead decision-makers, practitioners, or communities to overinvest or underinvest in resources in the most impactful or intertwined factors that could improve health and equity.

One potential way to focus attention on the most important interactions is to identify fundamental or root causes of health and equity, sometimes referred to as the “causes of the causes” or “upstream of the upstream.” Fundamental cause theory was developed by Link and Phelan^27^ who proposed four essential features for a fundamental social cause of health inequalities:

1. It influences multiple disease outcomes, meaning that it is not limited to only one or a few diseases or health problems.
2. It affects these disease outcomes through multiple risk factors.
3. It involves access to resources that can be used to avoid risks or to minimize the consequences of disease once it occurs.
4. The association between a fundamental cause and health is reproduced over time by the replacement of intervening mechanisms.

They believed that the reason for such persistent associations, and the essential feature of fundamental social causes, was that they involve access to resources that can be used to avoid risks or to minimize the consequences of disease once it occurs. They defined resources broadly to include money, knowledge, power, prestige, and beneficial social connections (social support and social networks). They have since suggested that there may be additional “fundamental causes” that include freedom, racism, discrimination, and stigma.^28,29^

Our examination of current frameworks for health and equity included in this study revealed several commonalities in the conceptual elements, such as community conditions and context, which have been increasingly understood as the social determinants of health. Interestingly, there was more variation in conceptual elements that extended health determinants into the realm of equity determinants. For example, only five frameworks included political or institutional power as drivers of health and equity and only two explicitly mentioned prejudice and stigma.

As noted above, nine of the frameworks identified some drivers as “fundamental” or “root” causes of health inequity. In addition to the fundamental causes suggested by Link and Phelan,^27^ Phelan and Link,^28^ and Hatzenbuehler et al.,^29^ other potential candidates identified from these frameworks include political, governance, and economic context; legal norms; and societal values. Several of the frameworks also added more specificity to the concept of power, including political and institutional power.

Notably, neither classic nor familiar models of the social determinants of health, such as Evans and Stoddart,^1^ the America's Health Rankings,^13^ and the County Health Rankings,^12^ were included because they include no explicit acknowledgment of equity. As Krieger^30^ pointed out, the determinants of health and the determinants of health inequalities are not necessarily the same. She argued for visual models that “clearly and unequivocally delineate the social facts of skewed distributions of power and resources and depict the societal processes that generate and maintain these distributions and their embodiment in population levels and distributions of health, disease, and well-being” (p. 1103).

The fact that causes identified as fundamental more than two decades ago^28^ are not consistently incorporated into our population health and equity frameworks has implications for our practice and research, and ultimately for our progress on persistent and in some cases growing health inequities. The variation across frameworks implies that the field of population health research and practice has yet to reach consensus on the determinants of health and equity and signals a nascent health equity field.

There are formative questions regarding the variation in and relationships among conceptual elements that could help the field move closer to consensus. To name a few, Are there additional criteria for fundamental causes beyond those offered in fundamental cause theory? Or criteria that distinguish determinants of health from determinants of equity? If health behaviors illustrate individual choices based on the opportunities we have, how does this fit into health and equity frameworks? And how can we measure complex overarching concepts, such as culture or power?

As noted in the introduction, this analysis does not represent an exhaustive catalog of frameworks and models of the drivers of health and equity nor is it designed as a formal evaluation of them. It also does not meet the strict criteria for a systematic review, for example, we did not record every framework that was derived from other frameworks (such as those from WHO CSDH^6^ or BARHII^7^) that we had already found. Specifically, although the image search proved helpful in locating additional frameworks, it captured many complete or partial duplicates. Other weaknesses of not having done a fully systematic search and review include a potentially biased selection of frameworks with a bias from the potential to omit, oversimplifying the content of frameworks, and the fact that for qualitative reviews, there is far more debate about what methods and approaches are appropriate.^31^

Another limitation beyond the challenges of obtaining a comprehensive set of frameworks was that our assessment of the frameworks was inherently subjective, although we tried to minimize this by using dual coding along with an independent arbiter for the tables where we summarized information from the frameworks. Our list of key elements was developed based on the criteria by the CCSDH^3^ and the criteria our organization's health equity work group had compiled when considering the desired attributes of a new framework. Thus, the list itself is subjective and may not be applicable to others who wish to develop new frameworks. For example, an anonymous reviewer questioned why we did not include asset-based approaches or issues of power and resources in our key elements. We did originally include asset-based approaches as a key element, but although some think this is desirable, asset-based approaches may not be able to appropriately acknowledge deeply entrenched drivers of inequity, such as historical trauma. So, in the end, we did not consider it to be “key.” Regarding issues of power and resources that we believe are fundamental drivers, our notion of key elements was based on potentially desirable characteristics of frameworks rather than as a list of specific drivers of health and equity.

Finally, although this article is about graphical depictions, we acknowledge that the article itself does not contain any pictures! Nor does the article focus on the graphical nature of the models (beyond simple characteristics such as showing interactions). Instead, our interest in this review was primarily on the content of the graphical depictions in terms of the types of drivers of health and equity that are presented.

## Conclusions

This review was conducted as part of an effort to develop a broader framework that reflects the drivers of health *and* the distribution of health within communities. We believe that this is a necessary step for the field of population health as we strive to be more equity focused and before identifying types of policies, systems changes, practices, and other actions that might improve health and equity outcomes. The range of action-oriented frameworks not captured in this analysis is also important and future work will be needed to minimize disconnects between driver-oriented and action-oriented frameworks.

Frameworks can be influential tools for advancing health and equity. However, the frameworks we examined were not consistent in their inclusion of the potential categories or dimensions of drivers of health and equity. As a result, they do not offer conceptual clarity on what shapes health and equity for the field of population health. This article highlights the variation in purposes, concepts, drivers, underlying theory or scholarly evidence, and accompanying measures put forth in current health and equity frameworks. Some variation is expected as it is difficult for any framework to be all things to all people. However, keeping in mind the importance of audience and purpose, the field of population health research and practice can work toward greater conceptual clarity on the drivers of health and equity. While numerous graphic representations of population health and equity exist, further work to visually represent underlying theories that drive value-based assessment of equity needs focused attention if we are to collectively advance our efforts for health and equity.

## Abbreviations Used

BARHII: Bay Area Regional Health Inequities Initiative
CCSDH: Canadian Council on Social Determinants of Health
CDC: Centers for Disease Control and Prevention
CO DPH: Colorado Department of Public Health
CRSIHS: Commission to Reduce Social Inequalities in Health in Spain
CSDH: Commission on the Social Determinants of Health
HEMF: Health Equity Measurement Framework
HI DOH: Hawai'i State Department of Health
NACA: National Advisory Council on Aging
NASEM: National Academies of Sciences, Engineering, and Medicine
NIA: National Institute on Aging
NIMHD: National Institute of Minority Health and Health Disparities
PAHO: Pan-American Health Organization
PHAC: Public Health Agency of Canada
RWJF: Robert Wood Johnson Foundation
SDH: Social Determinants of Health
SOPHIE: Structural Policies for Health Inequalities Evaluation
THRIVE: Tool for Health and Resilience in Vulnerable Environments
VA DOH: Virginia Department of Health
WHO: World Health Organization
WRHA: Winnipeg Regional Health Authority
